# A systematic review of the biomarkers associated with cognition and mood state in bipolar disorder

**DOI:** 10.1186/s40345-024-00340-z

**Published:** 2024-05-17

**Authors:** Anaid Pérez-Ramos, Cristina Romero-López-Alberca, Maria Hidalgo-Figueroa, Esther Berrocoso, Jose I. Pérez-Revuelta

**Affiliations:** 1 Barcelona Clinic Schizophrenia Unit, Hospital Clinic of Barcelona, Neuroscience Institute, Barcelona, Spain; 2 Centre for Biomedical Research in Mental Health (CIBERSAM), ISCI-III, Madrid, Spain; 3 Personality, Evaluation and Psychological Treatment Area, Department of Psychology, University of Cadiz, Cadiz, Spain; 4 Clinical Management of Mental Health Unit, University Hospital of Jerez, Andalusian Health Service, Cadiz, Spain; 5 Neuropsychopharmacology and Psychobiology Research Group, Psychobiology Area, Department of Psychology, University of Cadiz, Cadiz, Spain; 6 Biomedical Research and Innovation Institute of Cadiz (INiBICA), Research Unit, Puerta del Mar University Hospital, Cadiz, Spain; 7 Neuropsychopharmacology and Psychobiology Research Group, Department of Neuroscience, Faculty of Medicine, University of Cadiz, Cadiz, Spain

**Keywords:** Biomarker, Bipolar disorder, Cognition, Mood state, Psychiatry

## Abstract

**Background:**

Bipolar disorder (BD) is a severe psychiatric disorder characterized by changes in mood that alternate between (hypo) mania or depression and mixed states, often associated with functional impairment and cognitive dysfunction. But little is known about biomarkers that contribute to the development and sustainment of cognitive deficits. The aim of this study was to review the association between neurocognition and biomarkers across different mood states.

**Method:**

Search databases were Web of Science, Scopus and PubMed. A systematic review was carried out following the PRISMA guidelines. Risk of bias was assessed with the Newcastle–Ottawa Scale. Studies were selected that focused on the correlation between neuroimaging, physiological, genetic or peripheral biomarkers and cognition in at least two phases of BD: depression, (hypo)mania, euthymia or mixed. PROSPERO Registration No.: CRD42023410782.

**Results:**

A total of 1824 references were screened, identifying 1023 published articles, of which 336 were considered eligible. Only 16 provided information on the association between biomarkers and cognition in the different affective states of BD. The included studies found: (i) Differences in levels of total cholesterol and C reactive protein depending on mood state; (ii) There is no association found between cognition and peripheral biomarkers; (iii) Neuroimaging biomarkers highlighted hypoactivation of frontal areas as distinctive of acute state of BD; (iv) A deactivation failure has been reported in the ventromedial prefrontal cortex (vmPFC), potentially serving as a trait marker of BD.

**Conclusion:**

Only a few recent articles have investigated biomarker-cognition associations in BD mood phases. Our findings underline that there appear to be central regions involved in BD that are observed in all mood states. However, there appear to be underlying mechanisms of cognitive dysfunction that may vary across different mood states in BD. This review highlights the importance of standardizing the data and the assessment of cognition, as well as the need for biomarkers to help prevent acute symptomatic phases of the disease, and the associated functional and cognitive impairment.

**Supplementary Information:**

The online version contains supplementary material available at 10.1186/s40345-024-00340-z.

## Background

### Cognition in bipolar disorder

Bipolar disorder (BD) is a chronic psychiatry disease characterized by the recurrence of acute mood episodes with euphoric, depressive or mixed clinical features (Carvalho et al. 2020). Cognitive alterations negatively affect the disease course, the functional outcome in mood disorders, particularly in BD (Burdick et al. 2010; Mora et al. 2013; Torrent et al. 2012). Previous literature shows that BD patients’ present impairment in most cognitive domains (processing speed, declarative memory, executive function and attention) compared to healthy controls (HCs) (Li et al. 2020; Sanches et al. 2015). Despite this cognitive impairment being also present during remission phases (Bourne et al. 2013; Chen et al. 2023), there is considerable heterogeneity among patients with BD. This heterogeneity ranges from patients with intact cognition and performance comparable to HCs, to patients with significant global cognitive impairment (Burdick et al. 2014; Ehrlich et al. 2022), suggesting that there are different subgroups in function of cognitive performance. Studies examining the prevalence of cognitive impairment in BD report inconsistent results. A recent study examines the prevalence of cognitive impairment in a cohort of euthymic patients and estimates that 35% of patients experienced clinically significant cognitive deficits (Tsapekos et al. 2021).

In recent years, most studies have focused on euthymic patients’ cognition, while fewer compare it across both the acute and euthymic phases of BD. Research suggest it worsens during manic or depressive acute episodes (Kurtz and Gerraty 2009). For example, executive function (EFs) problems may arise early and tends to be exacerbated during depression and after manic episodes, suggesting it may be considered as a marker of the disease state (Elshahawi et al. 2011; López-Jaramillo et al. 2010). Supporting this, studies show worse cognitive performance in BD patients compared to HCs, with depression impacting working memory more than hypomania (Schouws et al. 2020) and manic episodes causing the most significant impairment across various cognitive domains (Vrabie et al. 2015). However, there are conflicting findings, with one study not detecting cognitive differences between depressive, manic and euthymic BD states (Martínez-Arán et al. 2004). This highlights the need for further research to understand how BD’s different phases influence cognitive function.

Accordingly, one of the main objectives in the management of psychiatric disorders would be to prevent or limit any cognitive deterioration by studying the factors involved in neurocognitive performance (Martínez-Arán and Vieta 2015), including both objective and subjective measures to asses’ cognition from a longitudinal perspective (Sanguinetti Czepielewski et al. 2023). Holistic and comprehensive treatment of BD requires both subjective and objective cognitive measures (Bonnín et al. 2024). Furthermore, there are no clinically available treatments with direct pro-cognitive efficacy in mood disorders (Miskowiak et al. 2017) and there is little understanding of the reasons why some patients with BD develop significant cognitive deficits, while others remain cognitively intact during the different affective phases of the illness.

### Factors influencing cognition in BD

Cognition can also be affected by various factors, such as clinical symptoms, age of onset, the incidence of psychosis and pharmacological treatments (Uluyol et al. 2020). The effect of medications on cognition has been a subject of debate, with some studies suggesting cognitive deficits during prolonged lithium treatment (Wingo et al. 2009), while others report no significant effects (Burdick et al. 2020). Similarly, antipsychotic medication has also been linked to poorer cognitive performance (Cullen et al. 2016). Additionally, a higher estimated Intelligence Quotient (IQ) before the onset of the illness is associated with a slower cognitive decline as people age (Tsapekos et al. 2021), and better cognitive performance in late adolescence was associated with a lower risk of BD (Hiyoshi et al. 2017).

BD has a high prevalence of psychiatric comorbidities. Research suggests that these comorbid conditions, diagnosed in over half of adult BD patients during their lifetime (Loftus et al. 2020), may contribute to cognitive impairment by influencing neurobiological pathways involved in mood regulation and cognitive function. Individuals with BD and substance use disorder (SUD) may have greater cognitive impairment compared to individuals with BD without SUD comorbidity (Gogia et al. 2022). Other studies suggest a high prevalence of overweight and obesity among patients with BD (Afzal et al. 2021). Obesity-related conditions are associated with systemic inflammation and insulin resistance, which have been linked to alterations in brain structure and function, particularly in regions involved in cognitive processes (Schmitt and Gaspar 2023). Furthermore, there is evidence of a possible negative effect of overweight on cognitive function (Restrepo Moreno et al. 2017; Yim et al. 2012), with an observed effect of higher body mass index (BMI) on lower cortical thickness (McWhinney et al. 2023). Therefore, addressing these comorbidities may be essential for improving cognitive outcomes in individuals with BD.

### Association between biomarkers and cognitive dysfunction in BD

Understanding the physiological and biological mechanisms underlying cognitive impairment in BD remains a challenge (Strawbridge et al. 2021). Identification of biomarkers has become a promising tool to guide diagnosis, predict clinical status, help understand the pathophysiology of mental disorders and inform treatment strategies. While current diagnostic criteria for mental disorders are based solely on clinical features and behavioral observations, with no substantial biological validation (Brückl et al. 2020), Frey et al. (2013) summarized BD-related biomarkers from genetic, peripheral, and neuroimaging biomarkers. In addition, ‘omics' technologies, have contributed to the rapid discovery of many potential biomarkers (García-Gutiérrez et al. 2020). Recent years have seen an increase in the number of studies focusing on the neural correlates of BD (Muneer 2020), however few studies have addressed whether there is an association between biological mechanisms and cognitive dysfunction in the different affective states of BD. Peripheral biomarkers, including different classes of cytokines such as IL-6 and tumor necrosis factor alpha (TNF-α), markers of oxidative stress, and markers of the innate immune system, have also been of interest to understand the basis physiological and consequences of BD (Strawbridge et al. 2021). However, whether blood-based biomarkers are a reliable source for assessing changes within the brain is an ongoing debate. In a recent review, Chaves-Filho et al. (2024) describe important findings in the different phases of BD. For example, they highlight changes in glutamate levels in regions such as the dorsolateral prefrontal cortex (DLPFC) or the anterior cingulate cortex (ACC) during manic and depressive phases. Similarly, they describe an increased systemic proinflammatory response, with hypomanic or manic episodes associated with higher serum levels of IL-8 and TNF- α compared to early depressive episodes.

Inflammatory markers and neuroimaging findings suggest potential associations with cognitive dysfunction. An association has been observed between the inflammatory state measured by C-reactive Protein (CRP) and cytokine levels in peripheral blood and cognitive impairment in patients with schizophrenia and BD (Misiak et al. 2018; Uluyol et al. 2020). Furthermore, patients with first-episode BD exhibited worse EFs and higher tumor necrosis factor receptor 1 **(**TNFR1) levels than HCs (Chen et al. 2020). Hence, there appears to be an association between inflammatory processes and executive dysfunction. It has also been observed that peripheral Brain Derived Neurotrophic Factor (BDNF) levels may contribute to cognitive deficits in patients with BD (Petersen et al. 2021).

Neuroimaging has revolutionized the diagnosis and treatment of neurological and psychiatric disorders, offering insights into biomarkers treatment response and personalized therapies (Yen et al. 2023). Some standard neuroimaging techniques used in cognitive neuroscience research include functional magnetic resonance imaging (fMRI), diffusion tensor imaging (DTI) and electroencephalography (EEG). They provide new opportunities to investigate functional and structural connectivity, mapping brain networks, decoding cognitive processes, and identifying neurological disease biomarkers (Yen et al. 2023). The abnormalities in various brain networks, such as the default mode network (DMN) or the cognitive control network (CEN), are likely to different neural circuits that intertwine to form the phenotype of BD. Thus, BD is associated with alterations in both frontal and posterior structures of the DMN, primarily in the prefrontal cortex (PFC), posterior cingulated and inferior parietal regions (Bi et al. 2022). A longitudinal neuroimaging study demonstrated changes in prefrontal regions across mood states in subjects with BD. BD patients in manic phase exhibited increased connectivity with the right middle frontal gyrus compared to HCs, whereas in depressed BD subject’s connectivity was increase with the right medial frontal gyrus and left middle frontal gyrus (Cerullo et al. 2012). Another study showed different brain activity patterns depending on cognitive impairment, those with poorer cognitive performance exhibit lower activity in regions associated with CEN and higher activity DMN, whereas cognitively preserved patients show minimal hypoactivity compared to HCs (Zarp Petersen et al. 2022).

Another prominent focus of attention on “hot cognition” has been emotional processing in patients with BD. These studies suggest that the neural circuits involved in emotion processing and regulation are altered, with a primary role attributed to the amygdale (Wu et al. 2024). Additional longitudinal studies collected in a review have reinforced the notion that chronic cortical abnormalities exist within the frontal networks regulating emotion in BD (Chaves-Filho et al. 2024).

Finally, the use of machine learning techniques has emerged as a promising tool to distinguish between BD and similar conditions, allowing for more personalized treatment based on measurable markers (Colombo et al. 2022). Advanced semi-supervised machine learning techniques can aid in the detection of inflammation subgroups based on accessible peripheral blood biomarkers or use machine learning algorithms (Alexandros Lalousis et al. 2023) and neuropsychological measures to identify patients with BD (Wu et al. 2016). Thus, these algorithms are ideal for assessing multifactorial disorders and estimating the probability of specific outcomes at the individual level, as suggested by the literature.

### Mood states in BD

The state of euthymia or remission is defined as the absence of criteria for major mood episodes according to the Diagnostic and Statistical Manual of Mental Disorders (DSM) or low ratings on mood questionnaires such as the Hamilton Depression Rating Scale (HDRS) and the Young Mania Rating Scale (YMRS) (Wang et al. 2015). Depression is characterized by a mood bias toward negative affect and loss of interest, while mania or hypomania is characterized by elevated, expansive, or irritable mood (Wu et al. 2024). Similarly, we find that approximately 40% of patients with BD, experience mixed episodes, defined as a manic state with depressive features, or manic symptoms in a patient with bipolar depression (Castle 2014).

In the literature we found that there seem to be differences and abnormal activation depending on mood (Brady et al. 2017; Keener and Phillips 2007; Sundaresh et al. 2018; Wu et al. 2024). Furthermore, despite an observed link between cognitive impairment in BD and neuroinflammation mechanisms (Bauer et al. 2014) and brain activity (Zarp Petersen et al. 2022), little is known about the biological events that underlie the cognitive deficits observed during the acute and euthymic phases of BD.

In this way, we hypothesize that there is an association between specific biomarkers and cognitive performance in different mood states of BD. This association may vary depending on the type of biomarker and the specific mood state.

Specifically, we expect to observe distinct patterns of association between biomarkers and cognition in depressive (BDD), manic (BDM), euthymic (BDE), and mixed (BDX) mood states.

The aim of this systematic review was to synthesize studies in the literature that evaluated the association between biomarkers and cognition in patients with BD according to affective state, since, to date, no study has systematically included these three factors.

## Methods

### Systematic search strategy

**Fig. 1:**
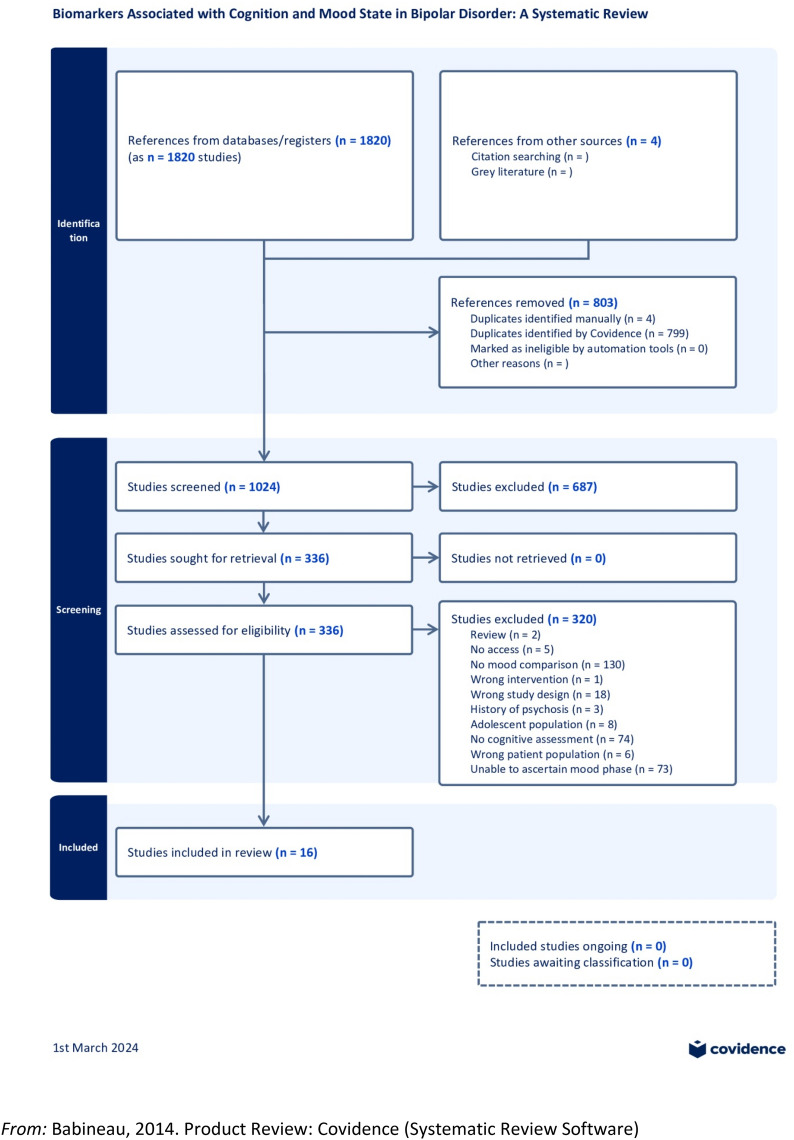
PRISMA flow diagram of the literature search and study selection. From: Babineau 2014. Product Review: Covidence (Systematic Review Software)

The study protocol was registered with the International Prospective Register of Systematic Reviews (PROSPERO) on 30th March, 2023 (Registration No.: CRD42023410782). Following the PRISMA guidelines (Preferred Reporting Items for Systematic reviews and Meta-analyses; Page et al. 2021), a systematic review was carried out of studies investigating biomarkers and cognition in the different mood states of BD (see Supplementary Material Appendix 1 for PRISMA Checklist). Searches of PubMed, SCOPUS and the Web of Science (WOS) were carried out for the past 10 years (from 2013), and we included studies of patients with BD in which data from at least two different mood states were compared. We started the search in August 2022 and concluded it in December 2022. To update the systematic review, a final search has been done to include those studies that could have been done during 2023. Figure 1 provides the methodological procedure followed was based on the PRISMA guidelines (Moher et al. 2009).

The syntax search [‘bipolar disorder’] AND [mood] AND [biomarker] AND [cogni*] in PubMed and its equivalents in the other databases were used. For the second search, studies from January 2023 to December 2023 have been included.

Note that the general term biomarker was used to include neuroimaging, physiological, genetic or peripheral markers.

### Eligibility

Eligibility criteria were: (a) the study was carried out on adult patients (≥ 18 years of age); (b) they were diagnosed with BD according to the criteria of the International Classification of Diseases (CIE-10) or the DSM (DSM-IV to DSM-5); (c) the study included a comparison of at least two phases of BD (mania, depression, mixed state or euthymia); (d) that includes a neurocognitive assessment (subjective and objective cognitive measures) and its association with a biomarker; (e) published from 2013 to December 2023 (e) longitudinal or cross-sectional studies and (f) it was written in English, Spanish or French.

The exclusion criteria were: (a) if the patients had a history of psychosis; (b) if the article was a family study, systematic review, book chapter, case report or a meta-analysis; (c) non-access articles.

### Data extraction

Two authors (CRLA and APR) performed the review independently using the Covidence program and any disagreements on study selection were resolved by a third person (JPR). Covidence is a web-based collaboration software platform that streamlines the production of systematic and other literature reviews (Babineau 2014).

### Data synthesis

The characteristics of the extracted studies are divided into two different tables. Table 1 gathers the studies that have examined peripheral biomarkers and cognition in different mood states of BD. Table 2 lists the various neuroimaging studies. The following characteristics were extracted from Tables 1 and 2: Author/year; Sample size; Sex; Mood state; Criteria to establish mood state; Biomarker; Cognitive assessment; Association between biomarker and cognition; Main Findings.

**Table 1:** Data extraction from studies on peripheral biomarkers and cognition in different mood states of BD

Author/year	Sample size (n)	Sex (m/f)	Mood state (n)	Criteria to establish mood state	Biomarker	Cognitive assessment	Association biomarker and cognition	Main Findings
1. Guidara et al. 2021	BD: 33 HCs: 40	BD: 33/0 HC: 40/0	BDD (6) BDM (27)	BDM moderate: MAS ≤ 21 BD	Tchol, 24-OCH, triglycerides, HDL-C, hs-CRP, LDL-C	MoCA	No correlation between the biological markers and cognition	Tchol < BDD 24-OHC < BDD CRP < BM
2. Idemoto et al. 2021	BD: 143 HCs: 158 MDD: 166	BD: 61/82 HC: 80/58 MDD: 80/78	BDD (58) BDE (58) BDM (7) BDX (19)	BDD: HAMD ≥ 8 and YMRS ≤ 7 BDE: HAMD ≤ 7 YMRS ≤ 7 BDM: HAMD ≤ 7 YMRS ≥ 8 BDX: HAMD ≥ 8 YMRS ≥ 8	Serum GDNF	Premorbid IQ with JART	No correlation between serum GDNF levels and cognition	Serum GDNF levels in BDD < HC IQ < HC

> higher, < lower, *HAMD* Hamilton Depression Rating Scale, *MAS* Bech and Rafaelsen Mania Scale, *YMRS* Young Mania Rating Scale, *BDM* bipolar mania, *BDD* bipolar depression, *BDE* bipolar euthymic, *BDH* bipolar hypomania, *BDX* bipolar mixed state, *MDD* major depressive disorder, *HCs* healthy control, *GDNF* Glial cell line-derived neurotrophic factor, *Tchol* Total cholesterol, *HDL-C* high-density lipoprotein cholesterol, *hs-CRP* high sensitivity C reactive protein, *LDL-C* low-density lipoprotein-cholesterol, 24-OCH cholesterol 24 hydroxycholesterol, *MoCA* Montreal Cognitive Assessment, *IQ* intelligence quotient, *JART* Japanese Adult Reading Test

**Table 2:** Data extraction from studies on neuroimaging biomarkers and cognition in different mood states of BD

Author/year	Sample size (n)	Sex (m/f)	Mood state (n)	Criteria to establish mood state	Biomarker	Cognitive assessment	Association biomarker and cognition	Main findings
3. Velasques et al. 2013	BD: 20 HCs: 12	BD: 14/6 HC: 3/9	BDD (10) BDM (10)	N/A Clinical Global Impression-Bipolar Version (CGI-BP)	EEG	Saccadic attention task	Gamma coherence varies according to the group and the area of the cortex observed	↓ Saccade latency in BDM < BDD and HC ↓ Frontal eye field Fz/F4 in BDM < BDD and HC ↑ FCr gamma coherence Fz/F8 in BDD > HC
4. Magioncalda et al. 2016	BD: 61 HCs: 42	BD: 18/43 HC: 15/27	BDD (20) BDE (20) BDM (21)	BDD and BDM: HAM-D 17 ≥ 18 and/or YMRS ≥ 13 BDE: HAM-D < 8 and YMRS < 8	DTI	CPT Verbal Fluency	WM alterations were associated with cognitive deficits. CPT total hits correlate with the mean FA and with the mean MD and RD values. Fluency prompted by letter showed a correlation with the mean FA, MD and RD values	Worse performance in CPT and Fluency in BDD and BDM < HC ↓ FA in BDD and BDM < HC ↑ MD, RD in BDD > HC
5. Mikawa et al. 2015	BD: 47 HCs: 28	BD: 20/27 HC: 11/17	BDD (30) BDE (25)	BDD: HAM-D > 7 and YMRS < 10 BDE: HAM-D ≤ 7 and YMRS < 10 at least 2 months	NIRS	VFT	Negative correlations between the increase in mean oxy-Hb levels induced by the VFT in the left temporal regions (channels 51–52)	↑ Left temporal regions in BDE > BDD ↓ Left temporal regions in BDE < HC
6. Nishimura et al. 2015	BD: 27 HCs: 12	BD: 18/9 HC:4/8	BDD (16) BDH (11)	The cutoff point for the YMRS was 4	NIRS	IQ VFT	Correlation between left DLPFC function and hypomanic symptoms	↓ DLPFC left (CH49) in BDD < BDM/HC ↓ VLPFC left in BD groups < HC Longitudinal: Left DLPFC/temporal regions/ FPC (symptoms were present) > (were absent symptoms)
7. Pomarol- Clotet et al. 2015	BD:114 HCs: 38	BD: 52/62 HC: 18/20	BDD (38) BDE (38) BDM (38)	BDD: HDRS ≥ 15 BDE: HDRS ≤ 8 and YMRS ≤ 6 at least 3 months BDM: YMRS ≥ 18	fMRI	n-back WAIS-II TAP	Reduced activation in the dorsal parietal cortex in both mania and depression during cognitive task	↓ Dorsal parietal cortesx in BDD and BDM < BDE ↓ DLPFC in BDM < BDE Failure of de-activation in the medial frontal cortex
8. Magioncalda et al. 2016	BD: 61 HCs: 42	BD: 18/43 HC: 15/27	BDD (20) BDE (20) BDM (21)	BDD and BDM: HAM-D 17 ≥ 18 and/or YMRS ≥ 13 BDE: HAM-D < 8 and YMRS < 8	DTI	CPT Verbal Fluency	WM alterations were associated with cognitive deficits. CPT total hits correlate with the mean FA and with the mean MD and RD values. Fluency prompted by letter showed a correlation with the mean FA, MD and RD values	Worse performance in CPT and Fluency in BDD and BDM < HC ↓ FA in BDD and BDM < HC ↑ MD, RD in BDD > HC
9. Martino et al. 2016	BD: 61 HCs: 42	BD: N/A HC: N/A	BDD (20) BDE (20) BDM (21)	BDD and BDM: HAMD ≥ 18 and/or score YMRS ≥ 13 HAM-D	Rs-fMRI DTI	CPT	Cognitive scores correlated with the measures of cingulum SC	BDD and BDM more omissions errors in CPT ↓ PACC-PCC functional connectivity in BDM < BDD and HC ↓ FA in BDM < BDE ↓ SC of the cingulum in BDM < HC
10. Rive et al. 2016	BD: 32 HCs: 35 MDD: 40	BD: 13/19 HC: 10/25 MDD: 12/28	BDD (9) BDE (23)	N/A	fMRI	IQ ToL	Task was associated with activity in parieto-temporal and lateral/medial frontal regions, in precuneus, insula, caudate nucleus and pallidum	↑ DLPFC in BDD > BDE
11. Lai et al. 2018	BD: 52 HCs: 31	BD: 26/26 HC: 15/16	BDD (30) BDE (22)	BDD: HDRS > 21 and YMRS < 7 BDE: HDRS < 8 and YMRS < 7 at least 6 months	MRI	TMT-B WCST	NAA/Cr ratio in left basal ganglia in the acute-episode was correlated with WCST and TMT-B uptake	Worse cognitive performance in BD group and NAA/Cr ratio in bilateral lenticular nucleus < HC
12. Alonso-Lana et al. 2019	BD: 26 HCs: 26	BD: 15/11 HC: 15/11	BDE (26) BDM (26)	BDE: YMRS and HDRS ≤ 8 BDM: YMRS ≥ 15 at least 2 months	fMRI	n-back TAP	Recovery from mania is associated with increase in activation in the left DLPFC/precentral cortex and the bilateral parietal cortex during n-back task	1-back and 2-back in BDM < BE Failure de-activation vmPFC in BD < HC ↓ Left DLPFC, PFC superior PaC in BDM < HC/BDE
13. Estudillo-Guerra et al. 2020	BD: 10 HCs: 10	BD: 2/8 HC: N/A	BDE (6) BDM(10)	BDE: MADRS < 6 and YMRS < 2 BDM: MADRS < 19 and YMRS > 20	SPECT	SCIP-S	Manic: positive correlation SCIP-S score and brain perfusion in the right TP. Negative correlation with right OFC and right sACC Follow-up: Brain perfusion was not correlated with SCIP-S	↑ Left DLFPC and left FPC in BDM > BDE
14. Yang et al. 2020	BD: 72 HCs: 71	BD: 32/40 HC: 33/38	BDD (32) BDE (25) BDM (15)	BDD: HAMD ≥ 17 and YMRS < 12 BDE: HAMD score < 17 and YMRS < 12 BDM: HAMD < 17 and YMRS ≥ 12	MRI	n-back	Increase in small-worldness was associated with decreased working memory accuracy	Worse working menory in BDD and BDM ↓ sigma and gamma in BDM < BDD ↓ Cingulo-opercular network in BDM and BDE < BDD
15. Gao et al. 2023	BD: 66 HCs: 60	BD: 36/30 HC: 38/22	BDE (28) BDM (38)	N/A	Rf-fMRI	PDQ	No correlation was found	↓ right IPL in BDM < BDD
16. Kopf et al. 2023	BD: 32 HCs: 31	BD: 29/22 HC: 10/20	BDD (32) BDE (15)	N/A	fNIRS	n-back	Right DLPFC activation during n-back task	No difference in DLPFC and vlPFC activation between BDD and BDE

↑ increased, ↓ decreased, > higher, < lower, *N/A* not applicable, *HAMD* Hamilton Depression Rating Scale, *HDRS* Hamilton Depression Scale, *MAS* Bech and Rafaelsen Mania Scale, *YMRS* Young Mania Rating Scale, *MADRS* Montgomery-Asberg Depression Rating Scale, *BDM* bipolar mania, *BDD* bipolar depression, *BDE* bipolar euthymic, *BDH* bipolar hypomania, *BDX* bipolar mixed state, *MDD* major depressive disorder, *HCs* healthy control, *FCr* right frontal cortex, *PaC* Parietal cortex, *PCr* parietal cortex right, *DLPFC* dorsolateral prefrontal cortex, *OFC* orbitofrontal cortex, *SACC* supragenual anterior cingulate cortex, *TPJ L* temporal parietal junction left, *TP* temporal polar cortex, *IPL* inferior parietal lobe, *PACC* perigenual anterior cingulate cortex, *PCC* posterior cingulate cortex, *vlPFC* ventrolateral prefrontal cortex, *MD* mean diffusivity, *RD* radial diffusivity, *FA* DTI-derived fractional anisotropy, *FCo* functional connectivity, *SPECT* brain perfusion single-photon emission computed tomography, *SC* structural connectivity, *WM* White matter, *NAA/Cr* N-acetylaspartate/creatine, *oxy-Hb* Relative concentration changes of oxygenated, *deoxy-Hb* concentration changes of deoxygenated, *rs-fMRI* resting-state functional magnetic resonance imaging, *MRI* Magnetic Resonance Imaging, *NIRS* near infrared spectroscopy, *fNIRS* functional near-infrared spectroscopy, *EEG* electroencephalography, *DTI* probabilistic tractographic diffusion tensor imaging, *TAP* Word Accentuation Test, *VFT* Verbal Fluency Test, *WAIS* Wechsler Adult Intelligence Scale, *IQ* intelligence quotient, *CPT* Continuous performance test, *SCIP-S* Screen for Cognitive Impairment in Psychiatry Scale, *ToL* Tower of London, *WCST* Wisconsin card sorting test, *TMT-B* Trail making test part B, *PDQ* Perceived Deficits Questionnaire

### Quality assessment

A quality assessment was carried out by APR and MHF using the Newcastle–Ottawa Quality Assessment Scale (Wells et al. 2000), rating each study in Table S2 (see Supplementary Material Appendix 2).

## Results

A total of 1824 articles were recovered for screening, of which 803 duplicates were removed and 687 were excluded as they did not deal with BD, while 4 articles were included as a result of the Snowballing effect (Wohlin 2014). Subsequent to review of titles and abstracts, 687 records were discarded and the full manuscripts of 336 studies were examined in detail. Of the articles included, only 16 explored an association between biomarkers and cognition in different affective states, most of which demonstrated a correlation between the cognitive functions evaluated and the different alterations during the mood phases of the disorder.

### Cognitive and biomarkers findings across affective state

Studies included markers from serum or plasma and neuroimaging. The studies were grouped into the following cognitive domains according to the cognitive tasks used used: "attention", "executive functions", "memory (working memory and verbal memory)", "IQ" "self-reported cognitive" and "Cognitive Screening Test ".

Fourteen studies used a combination of neuroimaging and neurocognitive assessments to investigate the affective states in BD (Alonso-Lana et al. 2019; Estudillo-Guerra et al. 2020; Gao et al. 2023; Kopf et al. 2023; Lai et al. 2018; Magioncalda et al. 2016; Magioncalda et al. 2015; Martino et al. 2016; Mikawa et al. 2015; Nishimura et al. 2015; Pomarol-Clotet et al. 2015; Rive et al. 2016; Velasques et al. 2013; Yang et al. 2020).

One study investigates attention using quantitative EEG parameters (qEEG), two studies employ resting-state functional magnetic resonance imaging (rs-fMRI), one study uses magnetic resonance spectroscopy (MRS), one study uses DTI, and there are five fMRI studies, among which one employs a technique to examine typology (connectome), along with two studies using the proton near-infrared spectroscopy (NIRS), one using functional near-infrared spectroscopy (f-NIRS) and one study performed Brain perfusion single-photon emission computed tomography (SPECT). Two studies used peripheral markers (Guidara et al. 2021; Idemoto et al. 2021). In these two studies, total cholesterol, triglycerides, high-density lipoprotein cholesterol (HDL-C), high sensitivity C-reactive protein (hs-CRP), low-density lipoprotein cholesterol (LDL-C), and serum Glial cell line-derived neurotrophic factor (GDNF) are used as measures of peripheral biomarkers.

Four studies used a longitudinal design (Alonso-Lana et al. 2019; Estudillo-Guerra et al. 2020; Kopf et al. 2023; Nishimura et al. 2015) and twelve were cross-sectional studies (Gao et al. 2023; Guidara et al. 2021; Idemoto et al. 2021; Lai et al. 2018; Magioncalda et al. 2016; Magioncalda et al. 2015; Martino et al. 2016; Mikawa et al. 2015; Pomarol-Clotet et al. 2015; Rive et al. 2016; Velasques et al. 2013; Yang et al. 2020).

#### Attention

Four studies carry out a neurocognitive evaluation of attention (Magioncalda et al. 2015, 2016; Martino et al. 2016; Velasques et al. 2013).

Three of them evaluate sustained attention with the continuous performance test (CPT; Magiocalda et al. 2016; Martino et al. 2016), where we find that BD patients showed lower number of total hits and higher number of total omission errors. On the one hand, BDM patients showed that structural changes in the cingulum were related to the deficits found at the attentional level. Furthermore, it was found that the perigenual anterior cingulate cortex (PACC) and posterior cingulate cortex (PCC) functional connectivity was decreased in BDM when compared to both HCs and BDD patients and the structural connectivity (SC) of the cingulum, especially its anterior part, was decreased in BDM when compared to HCs (Martino et al. 2016).

When microstructural abnormalities in the white matter (WM) were investigated using DTI neuroimaging technique, subgroups of BD patients showed different spatial patterns of WM alterations (Magioncalda et al. 2016). The BDE patients had minor and localized WM alterations in the midline structures, whereas the WM alterations were more diffuse in the BDM patients, affecting both midline and lateral structures, and there were stronger and more widespread WM alterations in BDD patients. In addition, these WM alterations were associated with attention deficits. Similarly, in another study these authors found differences in functional connectivity from the PACC to other regions in the posterior DMN between patients in manic or depressed episode and HCs, but no differences between the BD patient subgroups (Magioncalda et al. 2015).

Using qEEG, Velasques et al. (2013) found that BDM patients showed lower saccade latency than BDD patients or the HCs. In a prosaccadic attention task the BDM patients showed stronger gamma coherence in the frontal cortex than in the other groups (BDD and HCs).

#### Processing speed

Only one study evaluates processing speed (Estudillo-Guerra et al. 2020). Six months after an acute episode of mania, patients in euthymic state do not show differences in this cognitive sphere. At follow-up using SPECT technique, a decrease in perfusion was observed in the right middle temporal gyrus (MTG) and the right superior temporal gyrus (STG).

#### Executive functions

Seven studies explored EFs (Estudillo-Guerra et al. 2020; Lai et al. 2018; Magioncalda et al. 2016; Magioncalda et al. 2015; Mikawa et al. 2015; Nishimura et al. 2015; Rive et al. 2016). Three of them found no differences in performance between the groups (BD in different states and HCs) in the cognitive task (Mikawa et al. 2015; Nishimura et al. 2015; Rive et al. 2016).

Estudillo-Guerra et al. 2020 explored cognitive deficits in acute BDM patients and their subsequent evaluation after 6 months (euthymic state). This study evaluates cognitive functions using the Spanish version of the Screen for Cognitive Impairment in Psychiatry Scale (SCIP‐S). A subtest contains the Verbal Fluency Test (VFT) to evaluate EFs. A negative correlation between Brodmann area (BA) 25 and positive with BA 38 and 21 was found during a manic episode. At follow-up cognitive impairment in VFT correlated with changes increased perfusion in the bilateral ACC. Fluency prompted by letter showed a correlation with PACC and supragenual anterior cingulate cortex (SACC) (Magioncalda et al. 2015). By contrast, in another study with fMRI, there was increased activation in the dorsolateral prefrontal cortex (DLPFC) of BDD patients, and in the parietal cortex (PC) compared to the BDE patients (Rive et al. 2016). However, hypoactivation of the left DLPFC and of the left ventrolateral prefrontal cortex (VLPFC) during a VFT was found in patients with hypomanic symptoms, while this activation was less prominent in the DLPFC of BDD patients (Nishimura et al. 2015). In addition, this study followed hypomanic patients who showed significantly greater concentration changes of oxygenated hemoglobin (oxy-Hb) in the left DLPFC and frontopolar prefrontal cortex (FPPFC) when experiencing hypomanic symptoms compared to when they were absent. Similarly, the oxy-Hb levels induced by executive tasks were significantly lower in BDD than BDE patients (Mikawa et al. 2015). Finally, another study failed to find differences between the BD groups (Lai et al. 2018), showing a decrease in the N-acetylaspartate to creatine ratio (NAA/Cr) in the bilateral basal ganglia compared to the HCs. Nevertheless, the decrease in NAA/Cr ratios was negatively correlated with total errors and TMT-B uptake, but there was no correlation between the NAA/Cr in the right basal ganglia and the scores of WCST and TMT-B in acute-episode BD patients.

#### Memory

##### Working memory

Regarding working memory, four studies used an n-back paradigm (Alonso-Lana et al. 2019; Kopf et al. 2023; Pomarol-Clotet et al. 2015; Yang et al. 2020) and one used the SCIP-S subtest (Estudillo-Guerra et al. 2020). We found worse performance in BDM and BDD patients compared to HCs and BDE patients.

In a first study, the BDM group obtained worse results in the two versions of the n-back task compared to the BDD patients and HCs individuals (Pomarol-Clotet et al. 2015). However, when the cognitive load was increased (2-back version), the BDD patients also differed from the HCs. Surprisingly, the BDE patients did not differ from the HCs. There was reduced activation in the left and right dorsal PC and precuneus in BDM patients, and failure to de-activate the medial frontal cortex was evident in all BD groups.

In a longitudinal study using Brain Perfusion SPECT, patients were assessed during a manic episode and later, in a state of euthymia after about 12 months (Alonso-Lana et al. 2019). Similar to previous findings, BDM patients performed worse than HCs and BDE patients. Activation during the cognitive task showed weaker activation in the left DLPFC, PC, and bilateral superior precuneus in BDM patients, while the BDE group continued to exhibit failure in ventromedial prefrontal cortex (vmPFC) deactivation. During the working memory test of SCIP-S, manic episodes were associated with limited perfusion in the right orbitofrontal cortex (OFC), whereas no significant differences were observed during euthymia (Estudillo-Guerra et al. 2020).

Finally, functional neuroimaging data was used to provide an intuitive method to study fMRI-inferred neural efficiency in the whole brain, allowing interindividual differences related to the task to be predicted (connectome; Yang et al. 2020). An overall increase of the functional connectome was detected and there was a more homogeneous distribution in BDD patients. Interestingly, the maladaptive modulation of the functional connectome was associated with worse performance in working memory.

##### Verbal memory

Only one study assessed verbal memory (Estudillo-Guerra et al. 2020), with immediate verbal learning correlated to the temporal polar cortex. No significant correlation of manic episodes with delayed verbal learning was detected, although a significant correlation was seen in euthymic states.

#### Intellectual quotient

Concerning the IQ, HCs had a higher mean current IQ than the BDD and BDM patients but not the BDE patients (Pomarol-Clotet et al. 2015).

When the relationship between neurotrophic factors and cognition was studied in different mood phases of BD (Idemoto et al. 2021), no differences in plasma GDNF levels were evident between the affective states. Furthermore, no correlation was found between IQ and serum GDNF levels. However, after controlling for factors such as sex, age, BMI, estimated IQ, and diagnosis, serum GDNF levels in BD patients were lower in remission and depression states than HCs (this did not occur in patients in a manic or mixed state).

#### Cognitive screening test

Differences in the levels of oxysterols and CRP were analyzed in the distinct groups of BD, with lower cholesterol levels (Tchol, 24-OCH) reported in BDM patients relative to BDD patients and in patients with severe manic episode compared to those with moderate manic episode for 24-OCH levels (Guidara et al. 2021). By contrast, CRP levels were higher in BDM patients and in patients with severe manic episode compared to those with moderate manic episode. No correlations with the Montreal Cognitive Assessment (MoCA) were found.

#### Self-reported cognitive

A study utilizes a self-report measure (subjective cognition) to assess cognitive dysfunction with the Perceived Deficits Questionnaire (PDQ) and fNIRS (Gao et al. 2023). Despite finding differences in activation between patients in acute state and their remission state in the follow-up (BDM patients showed reduced network homogeneity compared to BDE), no association with cognition was found.

## Discussion

This systematic review represents an effort to synthesize the reliable evidence available on the associations between biomarkers and cognition in different phases of BD. When we look at these associations, we found a total of 16 articles that have addressed this issue.

### Acute mood episodes

#### Neuroimaging biomarker

Functional neuroimaging studies has highlighted the importance of modular and hierarchical brain networks for the functional integration of neural operations related to cognitive function (Park and Friston, 2013). Cognitive control and EFs are associated with activity in the PFC (Menon and D’Esposito 2022). Activation of the DLPFC, superior frontal gyrus, superior parietal lobule and precuneus are common neural correlates of working memory, EFs and attention (Friedman and Robbins 2022; Saldarini et al. 2022). Data from the *n*-back paradigm and fMRI studies suggest that there is a mood-state dependent hypoactivation in DLPFC and PC. In the included studies, during states of mania or depression there appears to be hypoactivation in the prefrontal and parietal cortex within the framework of a task that requires EFs or working memory (Bi et al. 2022; Brooks et al. 2015; Fleck et al. 2012; Penfold et al. 2015; Rodríguez-Cano et al. 2017; Takizawa et al. 2014). Although we found this hypoactivation also in the euthymic group (Saldarini et al. 2022), there could be less activation in frontal regions during the acute states of BD (Schumer et al. 2023). In fact, we found that moving from mania to euthymia was associated with an increase in activation in these areas (Strakowski et al. 2016). Interestingly, Peterson and colleagues (2021), found this hypoactivation in patients with poor cognitive performance, but after covarying for subsyndromal mood symptoms, it does not remain in DLPFC cluster in cognitively normal patients, which would imply that brain activity in the DLPFC region would be associated with cognitive performance, independently of sub-syndromic mood symptoms. Of note, functional neuroimaging research focused specifically on the mixed states of bipolar disorder is notably sparse (Wu et al. 2024).

However, a resting-state study observed reduced network homogeneity in the right inferior parietal lobe in patients with BDM compared to BDE (Gao et al. 2023). While this may serve as a potential biomarker for predicting mania remission status according to the authors, no correlations with cognitive tasks were found. This could be attributed to the nature of the task, as this region has been associated with language, social cognition, and other functions (Numssen et al. 2021).

On the other hand, DTI, a type of MRI used to visualize the WM tracts in the brain (Yen et al. 2023), has revealed WM abnormalities during all affective states of BD (Hu et al. 2020), being more prevalent in active phases of the disease. Thus, it seems that an acute mood state may be associated with acute state-dependent microstructural WM changes (Zanneti et al. 2009). BDD patients have the largest overall cluster size of WM alterations relative to BDE or BDM patients. Although no association between fractional anisotropy (FA) and antidepressants was evident in a meta-analysis (Favre et al. 2019), this could explain the difference between the alterations in the acute state, as other studies have found an association with treatment (Diego-Adeliño et al. 2014). Nevertheless, longitudinal studies would be better suited to identify and predict the effect of age, illness duration/severity and medication on WM microstructure in patients with BD (Favre et al. 2019).

Regarding MRS, different studies report conflicting results for the NAA/Cr ratio. Both an increase (Zhong et al. 2018) and a decrease in the NAA/Cr ratio were detected in the bilateral lenticular nucleus of BDD and BDM patients relative to the HCs (Frye et al. 2007). However, a correlation was found between the NAA/Cr ratio in the left basal ganglia in acute-episode BD patients and those with better EFs (Zhong et al. 2018). Similarly, other patterns of impaired functional connectivity have been proposed within dorsal attention networks that could differentiate mood states in BD, such as weaker connectivity in BDE patients and hyper-connectivity in BDM patients (Brady et al. 2017; Cerullo et al. 2012). A study using VFT found that BDD patients had weaker activation in both the right and left PFC than controls (Fu et al. 2018). In addition, patients show weaker activation for a second cognitive task (Tower of London test) in the bilateral DLPFC (Fu et al. 2018), although an increase in activity was described in the frontostriatal areas (Rive et al. 2016).

On the other hand, functional connectivity studies of the brain (functional connectome) show distinct patterns in specific neural networks in patients with different states of BD. An increase in the small world (functional connectome) was described in BDD patients, associated with worse performance in working memory (Yang et al. 2020). These results might reflect a compensatory effect to control excessive rumination in the DMN (Claeys et al. 2022) or compensatory activity required by patients in a more severe state of BD, as observed in other disorders (Xing et al. 2016).

Neuropsychological data support the differences found between patients in an acute state, who present worse cognitive performance (Ryan et al. 2012). Therefore, verbal memory, attention and EFs seem to be affected in manic states (Bourne et al. 2013; Kurtz and Gerraty 2009; Vrabie et al. 2015) and these deficits correlate with brain alterations (Benabarre et al. 2005; Pattanayak et al. 2012; Yamada et al. 2015). In contrast, deficits in working memory processing have also been consistently reported in euthymic patients (Thompson et al. 2007; Daglas et al. 2015) and no main effect of mood is found (Manelis et al. 2022). Therefore, differences have been seen in EFs and working memory in mania (Volkert et al. 2016), however finding differences between mania and euthymia may be due to a higher number of past manic episodes that were associated with poorer cognitive performance (Martínez-Arán et al.^ 2004^) or the history of psychosis (Allen et al. 2010; Simonsen et al. 2011), which we have tried to consider in this review.

#### Peripheral biomarkers

Regarding peripheral biomarkers, we see that lower cholesterol levels (Fusar-Poli et al. 2020) were reported in BDM patients relative to BDD patients, as well as higher CRP levels (Ekinci and Ekinci 2020; Tsai et al. 2017). However, other studies failed to find differences between the depressive and manic state (Sundaresh et al. 2018). Some studies have pointed towards an inflammatory component in BD, and it was suggested that elevated CRP levels might rather be a state than a marker in this condition (Evers et al. 2019; Fernandes et al. 2016). Although we found no correlations between cognitive variables and markers of inflammation here, serum CRP expression was negatively correlated with performance scores of immediate memory, language and attention in BD patients when the Repeatable Battery for the Assessment of Neuropsychological Status (RBANS) was used (Bauer et al. 2014).

The serum GDNF levels in BD patients in a mixed state showed no significant difference from those in HCs. Although altered levels of GDNF were only found in BDD patients, an increase in serum GDNF relative to the activity of the immune system occurred in BDM and BDD patients (Tunca et al. 2014), and there was no difference between BDE patients and HCs (Rosa et al. 2006). Moreover, the estimated IQ values, verbal memory and EFs of the BD mixed group were significantly lower than those of HCs (Vreeker et al. 2016). We have seen that GDNF levels in BDD patients decrease relative to those of the HCs (Takebayashi et al. 2006; Zhang et al. 2010), whereas those levels in BD patients in a mixed or manic state were comparable to those of the HCs. Conversely, GDNF plasma levels were higher in BDE patients relative to BDM patients and HCs (Barbosa et al. 2011). Similarly, serum GDNF increases in bipolar patients during acute manic and depressive episodes (Rosa et al. 2006). There is no clear relationship between GDNF and mood states, although GDNF mRNA expression may be increased by antidepressants or lithium (De-Paula et al. 2016; Sousa et al. 2011). An association between peripheral levels of GDNF and cognitive function was found in patients with major depressive disorder (MDD; Liu et al. 2022; Zhang et al. 2014), which could suggest that GDNF is a biomarker for both BD and MDD in depressive states (Zinchuk et al. 2022).

### Euthymic/remission states

#### Neuroimaging biomarker

In fMRI studies, de-activation failure has been reported in the vmPFC in BDD and BDM patients, persisting in remission (Fernández-Corcuera et al. 2013; Tian et al. 2020; Verdolini et al. 2023). This de-activation failure finding unique to BD may be core to the illness and akin to a trait mechanism not impacted by mood states.

A meta-analysis consistently found trait-related deficits in EFs and verbal memory in patients with BD (Bourne et al. 2013). Executive dysfunction was also evident in BDE patients in our systematic review and hence, EFs deficits in BD may persist across different mood states, both in acute episodes and the euthymic state (Bourne et al. 2013; Rosa et al. 2010; Volkert et al. 2016). However, we found here studies where the performance of euthymic patients is comparable to that of HCs, which could be due to the subtype of BD type I or II (Dittmann et al. 2008) or to cognitive heterogeneity within the sample. This highlights the need to differentiate subgroups by cognitive performance (Burdick et al. 2014). Concerning working memory, there are deficiencies in the manic or depressed state but not in euthymia. Indeed, most fMRI studies using an *n*-back paradigm suggested there were no significant differences in accuracy or reaction times between BDE patients and HCs (Cremaschi et al. 2013). However, elsewhere such deficits seem to persist during disease remission. (Oh et al. 2019; Srivastava et al. 2019; Volkert et al. 2016).

In euthymic state there seems to be parietal hypoactivation (Hajek et al. 2013) and normalization of DLPFC activation, which is mainly altered during manic episodes (Van der Schot et al. 2010).

Hypoactivation of the PFC in verbal fluency tasks has also been found (Yoshimura et al. 2014). Regarding MRS, during the euthymic state the NAA/Cr ratio in the bilateral lenticular nucleus was lower than in HCs (Kraguljac et al. 2012), although they did not exhibit changes in the NAA/Cr ratio in the temporal or parietal cortex (Brambilla et al. 2005).

In the context of cognitive performance, it is observed that BDE patients also achieve lower performance than HCs, and these differences seem to increase with task complexity (Volkert et al. 2016). Furthermore, despite dysfunction in brain circuits related to working memory in patients with BD, other intact systems may help overcome this deficiency (Cremaschi et al. 2013).

#### Peripheral biomarkers

Unlike what was found in the study included here, where the differences GDNF levels for patients in the euthymic state were only found after correction (Rosa et al. 2006), Barbosa et al. (2011) found higher GDNF levels in BDE compared to BDM patients. Other studies do not find differences in GDNF levels between euthymia and HCs (Tunca et al. 2014). The inconsistency of results could be due to type II error, and larger sample sized studies are needed. With growing evidence that inflammation contributes to cognitive impairment in several medical conditions, it is crucial to investigate this aspect in bipolar disorder. However, until now, the relationship between inflammatory markers and affective symptoms is not completely (Strawbridge et al. 2021).

## Conclusions

Findings about state-specific anomalies across different studies are difficult to compare or interpret, in part because of differences in paradigms and technique used. Our findings highlight core regions involved in BD that are not only mood-specific, but also observed across mood states. Although individuals are clinically in remission, they still show abnormalities in brain connectivity, but a state-dependent topology appears to exist in BD and there appear to be underlying mechanisms of cognitive dysfunction that may be different in the different mood states of bipolar disorder.

Consequently, this systematic review highlights the need for greater consistency in the use of staging models in BD research to standardize the results and identify biomarkers. Monitoring patients and verifying the most significant biomarkers could prevent the onset of acute episodes, and the functional and cognitive deterioration this entails. Similarly, it could allow a more precise differential diagnosis and improve the patient’s quality of life. As such, it is important to create a framework incorporating genetics, neuroimaging and cognitive sciences in order to refine the classification of mental disorders. A multidimensional approach combining peripheral and neuroimaging biomarkers may provide a more comprehensive understanding of cognitive deficits across affective states in BD.

The results obtained in this review demonstrate the importance of considering BD with its different characteristics and shows the need for further longitudinal studies, as there are insufficient studies on hypo/mania, mixed states and clinical comparisons. Examining individuals in different affective states is crucial to identify mechanisms dependent on traits or the current state of symptoms (state), allowing the study of disease mechanisms to develop improved methods of diagnosis and treatment.

## Limitations

This systematic review has several limitations. Firstly, the sample of patients that we found in most studies is small and this may be due to the difficulty of evaluating these patients in acute states.

Due to the heterogeneity in BD, we sought to be rigorous with the inclusion and exclusion criteria. For instance, as the impact of psychotic symptoms on cognition remains unclear, we excluded studies indicating patients had psychosis symptoms or if a history of psychosis was not included as a covariate. Conversely, most of the included studies do not seem to control this factor, potentially confounding the results.

Methodological heterogeneity within and between studies is an important limitation of the articles included in this review, as different modalities are used in neuroimaging studies.

The absence of consensus for defining euthymia and the definition of clinically significant impairment also imposes difficulties in both clinical practice and research. Although the duration of clinical remission has been associated with a significant improvement of residual symptoms, in this review, the range of duration to establish a euthymic state is from 2 to 6 months.

The effects of medication or comorbidities (obesity, SUD) are other factors to consider in the search for biomarkers and in neuropsychological assessments. An important limitation is also the cross-sectional nature of the studies available, along with the small samples. Furthermore, the longitudinal studies analyzed here experience significant loss of patients to follow-up, complicating interpretation.

## Future directions

The following systematic review could serve to create interventions that combine cognitive rehabilitation with biological treatments. It would be interesting to consider subsyndromal conditions and the presence of residual mood symptoms, since they could have a negative impact on certain cognitive spheres and the cognitive deficit in the euthymic state could change after controlling for these factors (Tsitsipa et al. 2015). More powerful longitudinal studies that follow patients across mood cycles will be crucial to clarify the relationship between neurocognitive impairment and mood. When employing cross-sectional designs, potential confounding factors such as disease subtype, mood state, psychotropic medication use, illness duration, and comorbidities should be carefully considered, as they may influence neuroimaging measures.

Additionally, neuroimaging studies reveal problem areas such as DLPFC in patients with mental disorders. To improve cognition, we could use non-drug techniques such as transcranial magnetic stimulation (TMS) or transcranial direct current stimulation (tDCS) targeting those target areas (Hyde et al. 2022). Integrating different neuroimaging techniques such as DTI with other imaging modalities such as functional magnetic resonance imaging (fMRI) or EEG could equally provide a comprehensive understanding of how structural connectivity correlates with existing brain networks.

On another front, ongoing research delves into how neurocognitive functioning differs between BD patients with and without psychosis experiences (Glahn et al. 2007). It's imperative to clarifying the definition of the BD patient subgroup and ensuring homogeneous clinical samples, including unmedicated BD patients and patients experiencing a first episode of mania or hypomania.

It should be noted that it would be interesting for future reviews to include digital biomarkers, since in recent years there has been an increase in studies in this area. Digital biomarker represents a new approach aimed at measuring the human behavior by using smartphones. Preliminary results suggest that passive data collection could be used as a potential alternative to standard neuropsychological assessments (Nguyen et al. 2023).

Finally, the integration of advanced semi-supervised machine learning techniques could be a compelling approach to stratify subgroups in BD based on mood, complementing and addressing the heterogeneity often found in clinical practice.

### Supplementary Information

Supplementary material 1.

## Data Availability

All data generated during this study are included in this published article (supplementary information files).

## Abbreviations

24-OCH: 24-Hydroxycholesterol
BD: Bipolar disorder
BDD: Patients with bipolar disorder in a depressed state
BDE: Patients with bipolar disorder in a euthymic state
BDM: Patients with bipolar disorder in a manic state
BDNF: Brain-derived neuorotrophic factor
BG: Basal ganglia
BMI: Body mass index
CEN: Cognitive control network
CPT: Continuous performance test
CRP: C-reactive protein
DLPFC: Dorsolateral prefrontal cortex
DMN: Default mode network
DSM: Diagnostic and statistical manual of mental disorders
DTI: Diffusion tensor magnetic resonance imaging
EEG: Electroencephalogram
EFs: Executive functions
FA: Fractional anisotropy
fMRI: Functional magnetic resonance imaging
fNIRS: Functional near infrared spectroscopy
FPPFC: Frontopolar prefrontal cortex
GDNF: Glial cell line-derived neurotrophic factor
GDNF: Glial cell-derived neurotrophic factor
HCs: Healthy subjects
HDRS: Hamilton depression rating scale
IL-6: Interleukin-6
IL: Interleukin
IQ: Intellectual quotient
MD: Mean diffusivity
MDD: Major depressive disorder
MoCA: Montreal cognitive assessment
MRS: Magnetic resonance spectroscopy
MTG: Middle temporal gyrus
NAA/CR: N-acetylaspartate to creatine ratio
NIRS: Near infrared spectroscopy
OFC: Orbitofrontal cortex
Oxy-Hb: Oxygenated hemoglobinOxyhemoglobin
PACC: Perigenual anterior cingulate cortex
PC: Parietal cortex
PCC: Posterior cingulate cortex
PFC: Prefrontal cortex
qEEG: Quantitative EEG parameters
RD: Radial diffusivity
Rs-fMRI: Resting State functional magnetic resonance imaging
SACC: Supragenual anterior cingulate cortex
SC: Structural connectivity
SCIP-S: Screen for cognitive impairment in psychiatry scale
SPECT: Single-photon emission computed tomography
STG: Superior temporal gyrus
Tchol: Total cholesterol
TMT-B: Trail making test part B
TNF- α: Tumor necrosis factor α
TNFR1: Tumor necrosis factor receptor 1
TNRF1: Tumor necrosis factor receptor 1
VFT: Verbal fluency test
VLPFC: Ventrolateral prefrontal cortex
vmPFC: Ventromedial prefrontal cortex
WCST: Wisconsin card sorting test
WM: White matter
YMRS: Young mania rating scale
